# Severe hypoxia exerts parallel and cell-specific regulation of gene expression and alternative splicing in human mesenchymal stem cells

**DOI:** 10.1186/1471-2164-15-303

**Published:** 2014-04-23

**Authors:** Xinyang Hu, Rongrong Wu, Lina A Shehadeh, Qing Zhou, Cizhong Jiang, Xin Huang, Ling Zhang, Feng Gao, Xianbao Liu, Hong Yu, Keith A Webster, Jian’an Wang

**Affiliations:** 1 Cardiovascular Key Lab of Zhejiang Province, Department of Cardiology, The Second Affiliated Hospital, College of Medicine, Zhejiang University, Hangzhou 310009, P.R. China; 2 Department of Medicine, Division of Cardiology, University of Miami Leonard M. Miller School of Medicine, Miami, Florida, USA; 3 Interdisciplinary Stem Cell Institute, University of Miami Leonard M. Miller School of Medicine, Miami, Florida, USA; 4 Vascular Biology Institute, University of Miami Leonard M. Miller School of Medicine, Miami, Florida, USA; 5 Department of Molecular and Cellular Pharmacology, University of Miami Leonard M. Miller School of Medicine, Miami, FL 33101, USA; 6 School of Life Sciences and Technology, Tongji University, Shanghai 200092, PR China

**Keywords:** Hypoxia, Microarray, Alternative splicing, Stem cell Niche

## Abstract

**Background:**

The endosteum of the bone marrow provides a specialized hypoxic niche that may serve to preserve the integrity, pluripotency, longevity and stemness of resident mesenchymal stem cells (MSCs). To explore the molecular genetic consequences of such a niche we subjected human (h) MSCs to a pO_2_ of 4 mmHg and analyzed global gene expression and alternative splicing (AS) by genome-exon microarray and RT-qPCR, and phenotype by western blot and immunostaining.

**Results:**

Out of 446 genes differentially regulated by >2.5-fold, down-regulated genes outnumbered up-regulated genes by 243:203. Exon analyses revealed 60 hypoxia-regulated AS events with splice indices (SI) >1.0 from 53 genes and a correlation between high SI and degree of transcript regulation. Parallel analyses of a publicly available AS study on human umbilical vein endothelial cells (HUVECs) showed that there was a strong cell-specific component with only 11 genes commonly regulated in hMSCs and HUVECs and 17 common differentially spliced genes. Only 3 genes were differentially responsive to hypoxia at the gene (>2.0) and AS levels in both cell types. Functional assignments revealed unique profiles of gene expression with complex regulation of differentiation, extracellular matrix, intermediate filament and metabolic marker genes. Antioxidant genes, striated muscle genes and insulin/IGF-1 signaling intermediates were down-regulated. There was a coordinate induction of 9 out of 12 acidic keratins that along with other epithelial and cell adhesion markers implies a partial mesenchymal to epithelial transition.

**Conclusions:**

We conclude that severe hypoxia confers a quiescent phenotype in hMSCs that is reflected by both the transcriptome profile and gene-specific changes of splicosome actions. The results reveal that severe hypoxia imposes markedly different patterns of gene regulation of MSCs compared with more moderate hypoxia. This is the first study to report hypoxia-regulation of AS in stem/progenitor cells and the first molecular genetic characterization of MSC in a hypoxia-induced quiescent immobile state.

## Background

The stem cell niche refers to a well-defined physiological compartment that includes cellular and acellular components and serves to integrate systemic and local signals to regulate the biology of stem cells (reviewed in [1,2]). Like other such niches, the bone marrow provides highly specialized and heterogeneous microenvironments that determine the self-renewal, multipotency, survival and migration of residing hematopoietic and progenitor cells including mesenchymal stem cells (MSCs). Recently oxygen tension (hypoxia) has been recognized as an important component of stem cell niches that exerts control over the proliferation, differentiation and pluripotency of resident cells [3-5]. The oxygen tension of the endosteum, a narrow compartment of the bone marrow directly adjacent to the bone, is less than 10 mmHg, while that of the sinusoidal cavity ranges between 30–60 mmHg; therefore bone marrow cells are subject to a gradient of hypoxia the severity of which depends on their location within the niche [3,6]. Studies of embryonic stem cells (ESCs) as well as induced pluripotential stem cells (iPSC) indicate that oxygen gradients control stem cell functions. Culture of ESCs under an aerobic pO_2_ of 160 mmHg causes spontaneous differentiation that is suppressed by more physiological pO_2_ within the range of 14–36 mmHg. Further reduction of pO_2_ to <10 mmHg, equivalent to the bone marrow endosteum suppresses both differentiation and proliferation of ESCs while retaining pluripotency [7,8]. Studies on bone marrow or adipose derived MSCs have shown similarly that moderate hypoxic culture equivalent to the central BM sinusoidal niche enhances proliferation and protects against senescence while more severe hypoxia may block proliferation and induce cell death [9-16].

Culture of MSCs under moderate hypoxia has been shown to modulate gene expression by HIF-1/2-dependent and independent mechanisms [8,17-21]. In addition to the predicted HIF-1α target genes such as those required for anaerobic metabolism (glycolytic enzymes, glucose transporters), cell cycle (p21, p53), and angiogenesis (VEGF), moderate hypoxia was shown to mediate increased expression of Oct4 and telomerase activity of human bone marrow MSCs [16,22,23]. When cultured under moderate hypoxia (20–40 mmHg O_2_), MSCs display enhanced proliferation and migratory activity that has been attributed to increased Akt phosphorylation, expression of c-MET, VEGF, chemokine receptors CXCR4 and CXCR1, and increased phosphorylation of focal adhesion kinase [10,18,19,24-26]. Suppression of stem cell differentiation by hypoxia has been linked to Notch pathway signaling wherein hypoxia promotes recruitment of HIF-1α to the Notch intracellular domain and subsequently to Notch-dependent promoters thereby enhancing their expression [27]. Recently, HIF-1α was shown to regulate MSC proliferation through the enhancement of TWIST expression, which down-regulates the E2A-p21 pathway, inhibits senescence and increases proliferation [28]. Oxygen tensions between 20 and 40 mmHg enhance proliferation and pluripotency of stem cells whereas tension below 10 mmHg (<1%) inhibit proliferation and may promote apoptosis [8-12,17-19].

Up to 95% of all human genes are alternatively spliced [29,30]. AS results in changes in composition of an mRNA produced from a given gene, brought about by changes in splice site choice and thence the production of proteins with different properties. AS is regulated by cell specific, developmental, and extracellular signal-regulated cues and pathways (Reviewed in [31]). Aberrant alternative splicing can cause disease and may contribute to cancer and neurodegenerative disease [32,33]. Exon arrays allow for detection and quantification of AS on a genome-wide scale. There are currently only 2 such reports of genome-wide analyses of hypoxia-related changes in pre-mRNA splicing. One identified Lama3 as a hypoxia-related splice variant in head and neck cancers [34]. Another analyzed the effects of hypoxia on AS in human umbilical vein endothelial cells (HUVECs) and identified multiple alternative splice events [35].

Here we investigated the effects of severe hypoxia on gene expression, exon splicing, and phenotype of human (h) MSCs. The results reveal for the first time unique sets of (severe) hypoxia-activated and repressed genes, many of which differ from those reported previously for more moderate hypoxia. We report for the first time a coordinate increase in expression of acidic keratins perhaps indicating a partial mesenchymal to epithelial (epidermal) transition (MET), a decrease in insulin/IGF-1 signaling with lower phosphor-Akt, and decreased expression of anti-oxidant-related genes that suggests lower metabolism and growth compared with aerobic culture. The expression of differentiation-related markers is consistent with enhancement of osteogenic and angiogenic pathways perhaps at the expense of myogenesis and adipogenesis. We also identify a novel set of hypoxia-regulated alternatively spliced transcripts in hMSCs. To our knowledge this is the first study to report on patterns of hypoxia-mediated alternative splicing in stem cells. The results provide a molecular framework for understanding the role of severe hypoxia in preserving bone marrow progenitor cell integrity and perhaps insights into the role of hypoxia in regulating cell biology in hypoxic niche environments such as the endosteum.

## Results

### Isolation and characterization of human MSCs

**Figure 1:**
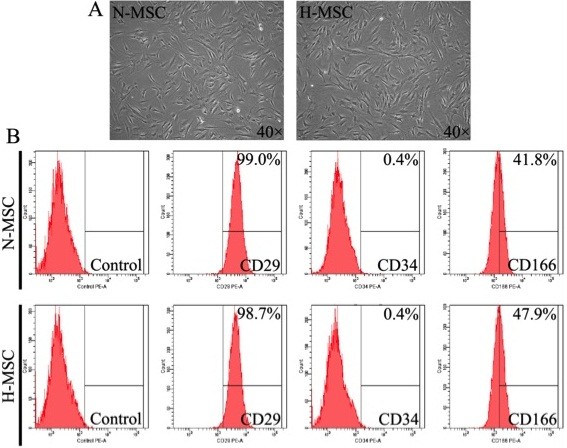
**Characterization of hMSCs cultures under air or hypoxia. (A)** Morphology and **(B)** surface antigen profiling of normoxic (N-MSC) and hypoxic hMSCs (H-MSC). Conditions and procedures are described in Methods. Representative of n = 3.

Human bone marrow MSCs were isolated as described in Methods and used at passage 8. At this time cells were visually homogeneous, fibroblast-like and positive for the expression of mesenchymal-specific markers CD29 (99.0%) and CD166 (41.8%) and negative for the expression of hematopoietic lineage marker CD34 (0.4%). These characteristics were unaltered after exposure to hypoxia for 24 h (Figure 1A and 1B).

### Gene expression array

Gene expression profiles comparing normoxia and hypoxia were obtained using Agilent Human 4 × 180 K Exon and 8 × 60 K-GE microarrays as described in Methods. Only the Exon arrays are described in the present analysis and the GE arrays were used for confirmation of some gene transcripts. Hierarchical clustering of the Exon arrays confirmed high reproducibility between samples (Additional file 1: Figure S1). A robust response to hypoxia was confirmed by quantifying HIF-1α-regulated transcripts. As shown in Table 1, multiple well-characterized HIF-1α-regulated genes were represented including carbonic anhydrase (>5-fold), metallothionein (>4-fold) and VEGF-A (>4-fold). Most of these genes have been reported previously in similar high throughput analyses of MSCs exposed to hypoxia in the range 1-5% [14,17-19]. Noteworthy in our analyses are the strong inductions of leptin and insulin-like growth factor binding protein 1 transcripts, confirmed in both Exon and K-GE arrays (latter data not shown) and relatively low induction of Bnip3 and glucose transporters. Glycolytic enzyme genes are widely recognized as markers of hypoxia with at least 8 of 11 glycolytic enzymes genes responsive through the HIF-1α pathway (reviewed in [36]). Surprisingly, glycolytic gene transcripts were not represented in the 2.5-fold cut-off groupings used for our array analyses, therefore we used RT-PCR to quantify phosphofructokinase (PFK) a major rate-limiting enzyme [37], phosphoglycerate kinase (PGK) a strong hypoxia responder [38], phosphoglycerate mutase (PGAM), a moderate responder [39] and glyceraldehyde-3-phosphate dehydrogenase (GAPDH), that is regulated by hypoxia in a tissue-specific manner [40-42]. As indicated in the Table, transcript levels of PFK and PGK increased, there was no significant change of GAPDH (p = 0.20, n = 7) and PGAM transcripts decreased (p = 0.02, n = 7). The results suggest mixed responses of individual genes. Glycolytic flux increased significantly under hypoxia; aerobic cultures used glucose at a rate of 2.83 ± 0.2 mg/dL/h compared with 3.33 ± 0.1 mg/dL/h for hypoxia (p > 0.05; n = 3), and there was a parallel increased rate of lactic acid generation over 24 h (data not show). Glycolytic flux is largely regulated by small molecule binding and allosteric control of PFK, the main rate-limiting enzyme and most of the pathway enzymes are present in large excess. Induction of gene expression by hypoxia is likely to be a chronic adaptation that is not required for the acute response to substrates and energy level.

**Table 1:** HIF-1 regulated transcripts

**Gene description**	**Gene symbol**	**Fold change HX/NX**
Leptin	LEP	74.3
Insulin-like growth factor binding protein 1	IGFBP1	8.93
Phopshoglycerate kinase	PGK	7.10*
Lysyl oxidase-like 4	LOXL4	6.5
Carbonic anhydrase IX	CA9	5.55
Metallothionein 3	MT3	4.78
Vascular Endothelial Growth Factor A	VEGFA	4.61
Carbonic anhydrase XII	CA12	4.62
Lysyl Oxidase	LOX	3.99
Basic helix-loop-helix family member 40	BHLHE40	3.97
Adrenomedullin	ADM	3.39
Placental Growth Factor	PGF	3.28
Phosphofructokinase	PFK	3.20*
Insulin-like growth factor binding protein 3	IGFBP3	3.10
Angiopoietin-like 4	ANGPTL4	2.88
BCL2/adenovirus E1B 19 kDa interacting protein 3	Bnip3	2.17
Lactate Dehydrogenase-A	LDH-A	2.10
Glyceraldehyde-3-Phosphate Dehydrogenase	GAPDH	1.48*
PhopshoGlycerate Mutase	PGAM	0.70*
Solute carrier family 2 (facilitated glucose transporter), member 1	SLC2A1 (GLUT 1)	2.01
Solute carrier family 2 (facilitated glucose transporter), member 3	SLC2A1 (GLUT 3)	2.11

Genes were identified from the gene expression array and all values are significant (p < 0.05, n = 3). For genes with (*), transcripts were quantified by QPCR using 3 different preparations of hMSCs run in duplicate; (PGK p < 0.001; PFK, p < 0.01; GAPDH, p = 0.21; PGAM, p = 0.02; all n = 7).

**Figure 2:**
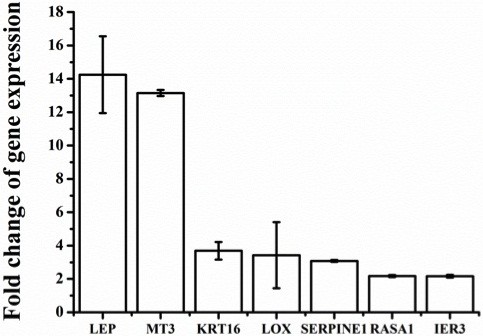
**Real-time PCR (RTqPCR) validation of differentially expressed genes.** RTqPCR was implemented on the same mRNA samples used for microarray as described in Methods. Results are mean ± SEM of n = 3.

**Figure 3:**
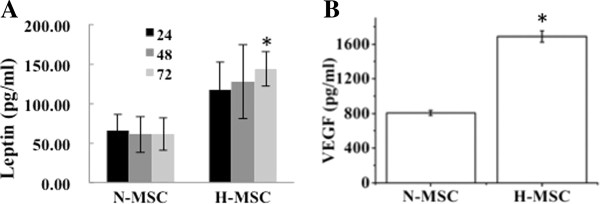
**Quantification of leptin and VEGF protein in MSC spent media during exposure to hypoxia. (A)** Numbers in left panel refer to time (h) under hypoxia. **(B)** VEGF incubation time was 24 h. All results are representative of 3 separate experiments; *p < 0.05, n = 3.

**Figure 4:**
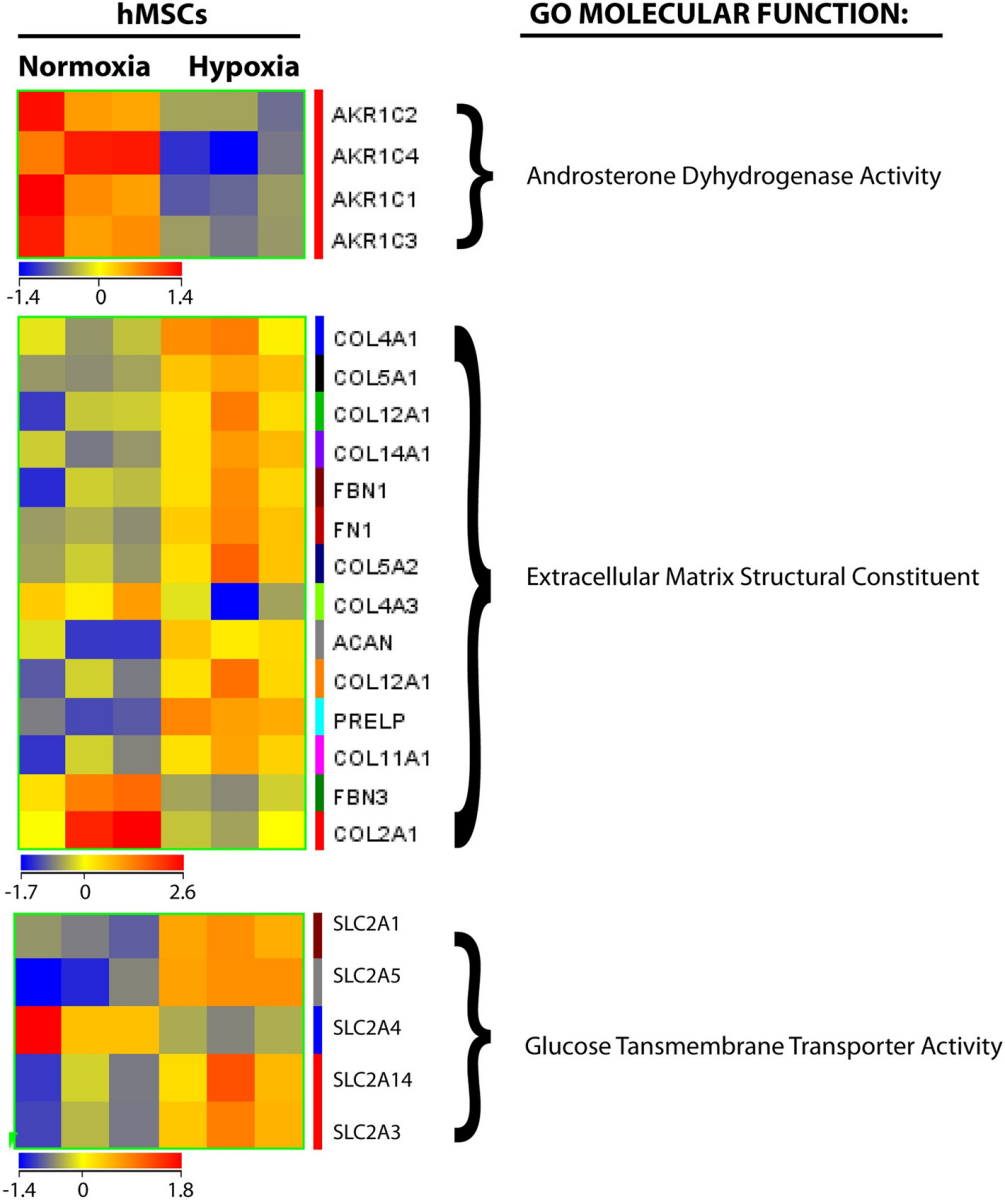
**Heatmaps of differentially expressed genes in 2 main Gene Ontology (GO) molecular functions.** Differentially expressed genes in human MSCs under hypoxia were subjected to GO analysis. A significance cut-off of p < 0.05 was used.

A total of 446 genes were found to have >2.5-fold change (p < 0.05). In agreement with previous reports on the effects of hypoxia on human MSC and endothelial cells [17-19,35] we found that more genes were down- than up-regulated (243 vs. 203). In order to confirm the microarray results RT-PCR was implemented on 3 known HIF-1α target genes in addition to the glycolytic transcripts described above, and four hypoxia-induced genes not previously identified as HIF targets. As shown in Figure 2, leptin (LEP), metallothionein-3 (MET3) and lysyl oxidase (LOX) were all confirmed to increase in a manner that reflected the array data. Similarly transcripts of non-HIF-regulated genes, keratin-16 (KRT16), Serpin peptidase inhibitor-1 (PAI-1), RAS p21 protein activator (RASA1) and Immediate Early Response 3 (IER3) were also confirmed to increase in the hypoxic samples in a manner that reflected the array data. Western blot and ELISA further confirmed that the levels of secreted hypoxia-marker genes VEGF and leptin were significantly increased by hypoxia in the spent media (Figure 3A and B). Figure 4 and Tables 2 and 3 show some of the most strongly hypoxia-regulated genes separated into functional categories. GO analyses identified 3 categories including [1] antioxidant pathways, NAD(P)H quinone-1, aldo-keto reductase family members and thioredoxin reductase were markedly down-regulated [2] extracellular matrix structural constituents in particular collagens were subject to up and down regulation suggesting dynamic changes of the ECM and [6] glucose transporters that were also subject to both up and down-regulation (see Figure 4 and Tables 2 and 3).

**Table 2:** Up-regulated genes and functional group allocations

**Class**	**Gene description**	**Gene symbol**	**HX/NX**
**Differentiation**			
	Leptin	LEP	74.3
	Early Growth Response 2	EGR2	6.78
	Inhibin, beta B	INHBB	6.25
	Interleukin 11	IL11	5.5
	Growth differentiation factor 6	GDF6	5.29
	Hemopoietic cell kinase	HCK	4.82
	Podoplanin	PDPN	3.65
	Ephrin-A3	EFNA3	3.65
	Semaphorin A7	SEMA7	2.70
	Activin A receptor type IIA	ACVR2A	2.70
	Matrix metalloproteinase 11	MMP11	2.52
	Aggrecan	ACAN	2.50
	Sparc/osteonectin (testican)	SPOCK1	2.2
	Frizzled homologue-8	FZD8	3.2
	Secreted frizzled related protein 4	SFRP4	2.3
	Cadherin 11, type 2 (osteoblast)	CDH11	2.0
**Survival/Apoptosis**			
	Transient receptor potential cation channel	TRPM7	6.63
	Interleukin 11	IL11	5.5
	Metallothionein 3	MT3	4.80
	Stanniocalcin 1	STC1	4.8
	Regucalcin	RGN	4.04
	Vascular endothelial growth factor A	VEGFA	3.56
	RAS p21 protein activator 1	RASA1	2.92
	Immediate Early Response 3	IER3	2.52
**Proliferation/Survival**			
	Insulin-like growth factor binding protein 1	IGFBP1	8.93
	Insulin-like growth factor binding protein 3	IGFBP3	3.1
	Insulin-like growth factor binding protein 5	IGFBP5	3.1
	Interleukin 11	IL11	5.5
	Placental Growth Factor	PGF	3.28
	Transcription elongation factor A3	TCEA3	2.82
	Hepatocyte growth factor receptor	MET	2.70
	Heparin-binding EGF-like GF	HBEGF	2.64
	Jun B oncogene	JUNB	3.07
	c-Jun oncogene	JUN	2.66
	Inhibin, beta B	INHBB	6.25
	Growth differentiation factor 6	GDF6	5.29
	Inhibitor of growth family member 3	ING3	3.53
**ECM migration/adhesion/cytoskeleton**			
	Early growth response 2	EGR2	6.78
	Interleukin 11	IL11	5.5
	Activated leukocyte cell adhesion molecule	ALCAM	2.89
	Junctional adhesion molecule 2	JAM2	3.10
	Semaphorin 5A	SEMA5A	4.02
	Connective tissue growth factor	CTGF	2.40
	Protein tyrosine phosphatase, receptor type, F	PTPRF	2.66
	Signal-induced proliferation-associated 1 like 1	SIPA1L1	4.0
	Intergrins alpha- 1, 3, 5, 6, 7	ITGA	>2.2
	Integrin beta-1	ITGB	2.1
**Intermediate filament**			
	Periplakin	PPL	7.88
	Desmoplakin	DSP	3.86
	Keratin 16	KRT16	6.39
	Keratin 14	KRT14	4.04
	Keratin 20	KRT20	4.40
	Keratin 19	KRT19	3.5
	Keratin 15	KRT15	3.35
	Keratin 13	KRT13	3.37
	Keratin 24	KRT24	3.09
	Keratin 17	KRT17	2.70
	Keratin 12	KRT12	2.60
	Mucin 1	MUC1	2.23
	KIAA1199	KIAA1199	5.2
	RAS p21 protein activator	RASA1	2.92
	Microtubule-associated protein 1B	MAO1B	2.70
	Microtubule-actin cross-linking factor 1	MACF1	2.4
	Lysyl oxidase-like 4	LOX4	6.5
	Lysyl oxidase-like 2	LOX2	2.5
**Angiogenesis**			
	Leptin	LEP	74.3
	Stanniocalcin 1	STC1	4.70
	Vascular endothelial growth factor A	VEGFA	4.61
	Placental growth factor	PGF	3.28
	Hepatocyte growth factor receptor	MET	2.70
	PDGF receptor B	PDGFRB	2.1
	PDGF receptor A	PDGFRA	2.0
	Angiopoietin-like 4	ANGPTL4	2.80
	Serpin peptidase inhibitor member 1	SERPINE1	4.92
	Serpin peptidase inhibitor member 2	SERPINE2	2.4
	Tissue plasminogen activator	PLAT	2.3
	Endothelial tyrosine kinase, Ang 1 receptor	TEK	5.16
	Lysyl oxidase-like 4	LOX4	6.5
	Desmoplakin	DSP	3.86
	Noggin	NOG	3.91
**Glucose Transport**			
	Glucose transporter member 1	SLC2A1	2.01
	Glucose transporter member 3	SLC2A3	2.11
	Glucose/fructose transporter member 5	SLC2A5	2.0
	Glucose transporter, member 14	SLC2A14	2.88

Genes were identified from the gene expression array using Genespring software and GO analysis to assign functional categories as described in Methods and manually from inspection of the arrays. All values are significant (p < 0.05, n = 3).

**Table 3:** Down-regulated genes and functional group allocations

**Class**	**Gene description**	**Gene symbol**	**HX/NX**
**Antioxidant**			
	NAD(P)H dehydrogenase, Quinone 1	NQO1	−5.63
	Aldo-Keto Reductase 1C1	AKR1C1	−2.71
	Aldo-Keto Reductase 1C2	AKR1C2	−2.61
	Aldo-Keto Reductase 1C3	AKR1C3	−2.60
	Aldo-Keto Reductase 1C4	AKR1C4	−4.28
	Aldo-Keto Reductase1D1	AKR1D1	−3.04
	Aldo-Keto Reductase 1B10	AKR1B10	−3.07
	Aldo-Keto Reductase 1B15	AKR1B15	−2.10
	Glucose 6 phosphate DH	G6PDH	−2.00
	Thioredoxin reductase	TXNRD1	−3.77
**Proliferation/growth associated**			
	Insulin-like growth factor 1	IGF-1	−3.2
	PI3-kinase regulatory subunit 2	PIK3R2	−2.9
	Platelet derived growth factor receptor-like	PDGFRL	−2.85
	Fibroblast growth factor 7	FGF7	−2.1
	Cell cycle arrest checkpoint	RAD9B	−4.4
	Mediator of DNA damage checkpoint 1	MDC1	−3.39
	Helicase (DNA) B	HELB	−4.4
	Inhibitor of DNA binding 1	ID1	−3.26
	Inhibitor of DNA binding 2	ID2	−3.0
	Inhibitor of DNA binding 4	ID4	−3.0
	TNF superfamily, member 14	TNFSF14	−2.22
**Migration-associated**			
	Phosphatidylinositol-3,4,5-trisphosphate-dependent Rac exchange factor 1	PREX1	−2.23
	Coronin 7	CORO7	−5.10
	Formin homology 2 domain containing 1	FHOD1	−2.15
	Actin filament associated protein 1 like 2	AFPAP1L2	−3.3
	Palmdelphin	PALMD	−2.8
	Tubulin, alpha 3D	TUBA3D	−3.9
**Glucose metabolism**			
	Glucose 6 phosphate DH	G6PDH	−2.00
	Pyruvate dehydrogenase (liver RBC)	PKLR	−2.61
	Phosphoglycerate mutase 2	PGAM2	−3.00
	Phosphoglycerate mutase 5	PGAM5	−2.27
	Phosphogluconate dehydrogenase	PGD	−2.55
	Glucose transporter, member 4	SLC2A4	−2.7
	Glucose transporter, member 8	SLC2A8	−2.5
	Glucose/fructose transporter, member 11	SLC2A11	−2.24
**Muscle/myogenesis/structural**			
	Actin alpha-1 (Sk)	ACTA1	−4.33
	Actin gamma-2 (SM)	ACTG2	−5.83
	Fer-1-like 5 (myotube formation)	FER1L5	−5.30
	Tripartite motif containing 16-like	TRIM16L	−4.95
	Myosin heavy chain 2	MYH2	−4.0
	Myosin heavy chain 7	MYH7	−3.0
	Troponin T type 2 (cardiac)	TNNT2	−2.8
	ATPase calcium channel (cardiac)	ATP2A1	−2.94
	Myosin binding protein B (fast)	MYBPC2	−2.79
**Miscellaneous**			
	Lysine acetyl transferase 2A	KAT2A	−3.45
	Galactosidase beta-1-like (senescence)	GLB1L3	−3.0
	Eukaryotic translation initiating factor 2B subunit gamma 3	EIF2B3	−2.6
	Eukaryotic translation elongating factor 1 epsilon 1	EEF1E1	−2.67
	Osteocrin	OSTN	−2.3
	Elongation factor RNA Pol II, 2	ELL2	−2.0
	Telomerase associated protein-1	TEP1	−2.67
	Death associated protein kinase 2	DK2	−2.4
	Protein kinase C delta	PRKCD	−3.37
	Interleukin-8	IL8	−3.05
	Interleukin-19	IL19	−2.61
	Interleukin-27	IL27	−3.30
	Interleukin-28A	IL28A	−2.24
	Chemokine (C-C motif) ligand 2	CCL2	−3.19

Genes were identified from the gene expression array using Genespring software and GO analysis to assign functional categories as described in Methods; additional genes were identified manually from inspection of the arrays. All values are significant (p < 0.05, n = 3).

### Intermediate and cytoskeleton filaments

**Figure 5:**
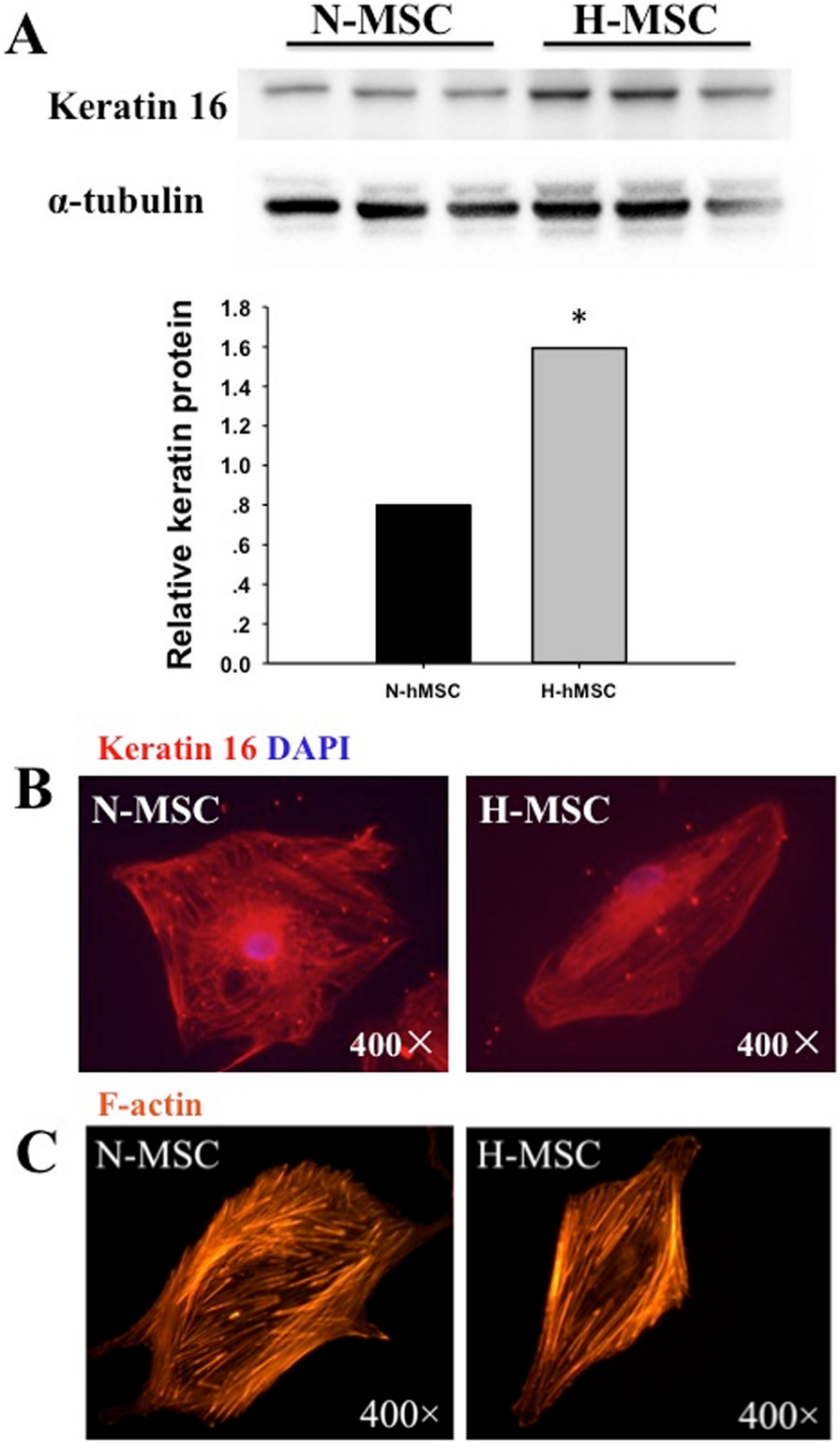
**Keratin induction and reorganization of intermediate- and micro-filaments under hypoxia. (A)** Western blot of Keratin-16 expression in normoxic (N-MSC) and hypoxic (H-MSC) human MSCs. **(B)** Keratin immunostaining and **(C)** F-actin immunostaining of MSCs cultured for 24 h under normoxia (N-MSC) or hypoxia (H-MSC). Western blots and immunostaining are described in Methods. All results are representative of 3 separate experiments; *p < 0.05, n = 3.

Intermediate filaments and microfilaments provide scaffolding that allows cells to rapidly remodel in response to environmental or intracellular signals, for example to increase or decrease movement and/or cell adhesion [43,44]. Intermediate filaments and their keratin structural components are markers of epithelium where in combination with cell junction components, desmosomes, hemidesmosomes and integrins, they contribute to the adhesion of epithelial cells to the basement membrane [43]. The keratins that compromise intermediate filaments are typically selected from 12 acidic (type 1) and 8 basic (type 2) cytokeratins that usually dimerize with specific partners from the opposite group to form organized filaments. Acidic keratins are coded on chromosome 17q whereas the basic keratins are clustered on chromosome 12q. The gene expression analysis revealed a remarkable increase in the expression of 9 out of 12 acidic keratins and decreased transcript levels of 3 (K2, K4, K5) out of 8 basic keratins with no change of the other members (Tables 2 and 3). To determine whether these changes in gene expression were reflected at the protein and cell structural levels, we measured K16 protein by western blot and the organization of intermediate and microfilaments respectively by keratin and F-actin immunostaining. As shown in Figure 5, K16 levels were increased by 2 ± 0.1-fold (p < 0.05) in MSC after exposure to hypoxia and this was associated with a change in the organization of intermediate filaments and altered cellular morphology (Figure 5B). The changes in cell shape and organization of cytoplasmic filaments indicated by keratin staining were also reflected by similar cell images imaged by F-actin immunostaining (Figure 5C). Other proteins related to intermediate filaments structure and function that were also increased by hypoxia include periplakin and desmoplakin, junctional adhesion molecule 2 (JAM2), adipocyte-specific adhesion molecule (ASAM) adhesion molecule with Ig-like domain 2 (AMIGO2) and podoplanin (PDPN) (see Table 2). In addition, six integrins (α-1,-3,-5,-6,-7, β-1) were increased by >2-fold. These changes may reflect a partial mesenchymal to epithelial transition (MET) that is a reversal of epithelial to mesenchymal transition (EMT) a well-established pathway associated with development and carcinogenesis. MET is predicted to involve reduced cell mobility and proliferative quiescence [45]. Interestingly hypoxia also increased the transcript levels of the HGF receptor c-MET that is normally expressed by cells of epithelial origin (Table 2).

### Regulation of differentiation

MSCs are pluripotent with the capacity to differentiate into adipocytes, osteocytes, chondrocytes, endothelium, neurons and muscle as well as hematopoietic cells. It has been reported that moderate hypoxia >10 mmHg increased [19,46] or decreased [23,47,48] adipogenic differentiation and increased [10,19,47,48] or decreased [20,23] osteogenic differentiation of MSCs. In our studies, combined GO and manual analyses revealed trends of hypoxia-regulated differentiation markers that suggest repression of adipogenesis and myogenesis in favor of osteogenesis, angiogenesis and hematopoiesis. Leptin, interleukin-11 (Il-11), growth differentiation factor-6 (GDF6), and MMP11, all strongly induced by hypoxia, are secreted factors that favor osteogenesis over adipogenesis [49-51]. Collagens are major constituents of the bone matrix and are highly expressed in MSCs. Type 1 collagen, the main osteogenic collagen was not amongst the regulated genes, however, multiple other osteogenic markers genes including aggrecan (ACAN), sparc/osteonectin (SPOCK1), osteoblast cadherin (CDH11) and Wnt pathway members Frizzled homologue-8 (FZD8) and Secreted frizzled related protein 4 (SFRP4) were induced in parallel with decreased expression of the negative osteogenic regulator osteocrin (Tables 2 and 3) [52,53]. Four of the most strongly induced genes including early growth response 2 (EGR2 > 6-fold) [54], interleukin 11 (Il-11 > 5-fold), growth differentiation factor 6 (GDF6 > 5-fold) and hemopoietic cell kinase (HCK ~5-fold) as well as activin-A receptor (2-fold [55]) are associated with hematopoiesis. Transcript levels of multiple genes associated with angiogenesis were increased by hypoxia [56] while there were marked decreases of multiple striated muscle markers (Tables 2 and 3). The coordinate decrease of 3 Inhibitor of DNA binding factors ID1, ID2, and ID4 (Table 3) is also consistent with a switch in differentiation patterns. Taken together the results support inductions of multiple marker genes for osteogenesis, angiogenesis, and hematopoiesis by severe hypoxia that may predispose cells to these lineages.

### Proliferation and metabolism

**Figure 6:**
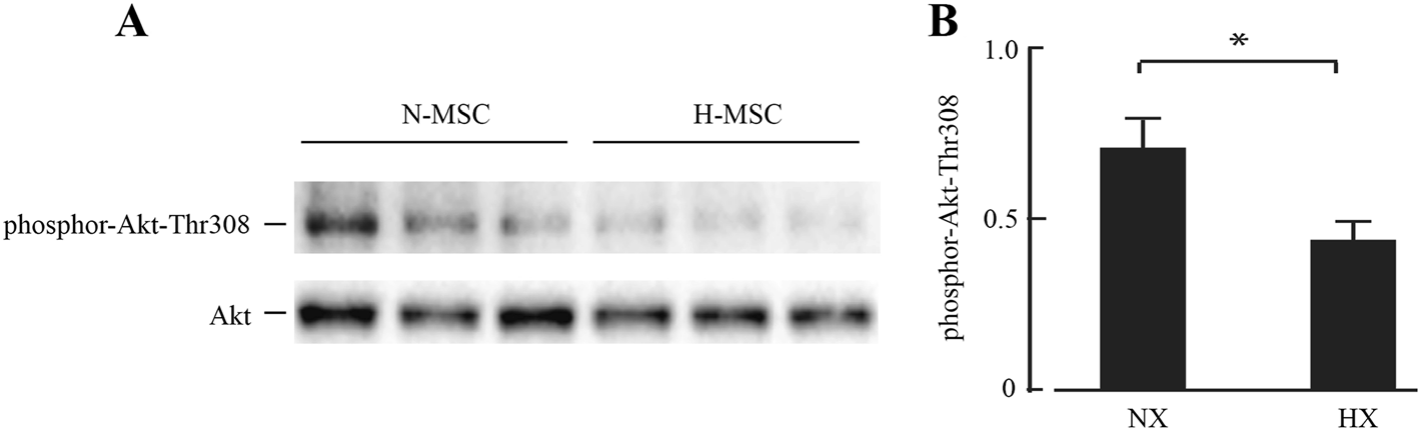
**Western blot and quantification of phosphor-Akt expression in normoxic (N-MSC) and hypoxic (H-MSC) human MSCs (A and B).** Western blot procedures are described in Methods. Akt-P-Thr308 quantification was by NIH image using total Akt as loading control; *p < 0.05, n = 3.

As discussed above, oxygen tensions above 15 mmHg enhance proliferative and migratory potential of MSCs while tensions below10 mmHg decrease proliferation [8-12,17-19]. In agreement with this we found that proliferation of hMSCs was decreased under a pO_2_ of 4 mmHg and the cultures became stationary after 3 days (data not shown). Enhanced proliferation under moderate hypoxia has been attributed to down-regulation of the p21-Ras pathway and increased activity of PI3-kinase-Akt while increased migration was attributed to increased expression of c-MET, VEGF, CXCR4 and CXCR1 (reviewed in [16]). Whereas we also observed increased expression of c-MET and VEGF-A we found no change in expression of CXCR receptors, increased expression of p21-Ras and decreased activity of the PI3-kinase-Akt pathway (see Tables 2 and 3). PI3-kinase-Akt is a central regulator of cell growth and survival. We found that the expression of both IGF-1 and PI3-kinase was significantly decreased under hypoxia while multiple IGF-1BPs were increased (Tables 2 and 3). To determine whether these changes were reflected by parallel changes in pathway activity we measured the phosphorylation of Akt-Thr308 after culture under normoxia or hypoxia. As shown in Figure 6, phosphor-Akt-Thr308 levels were significantly lower after hypoxia. This contrasts with the effects of moderate hypoxia where Akt phosphorylation is increased [26]. Other down-regulated transcripts related to growth and survival included senescence marker galactosidase beta-1-like (−3.0) and death associated kinase-2 (−2.4). There were also decreases of several interleukins and the cell migration cytokine CCL2, also known as MCP-1.

### Glucose metabolism

During hypoxic exposure, cells switch from oxidative metabolism to anaerobic glycolysis for energy production. Glycolysis is less efficient than oxidative phosphorylation and more glucose is required to sustain the same level of cell function. Glycolytic enzyme and glucose transporter genes are regulated by HIF-1α and previous studies have reported their induction by moderate hypoxia in the range of 20–40 mmHg [13,14,17-19]. We found increased transcript levels of at least 2 key glycolytic pathway genes, PFK and PGK, decrease levels of PGAM, no change of GAPDH and decreased transcripts of glucose-6-phosphate dehydrogenase (G6PDH) and pyruvate dehydrogenase PDH; the latter two enzymes are involved in the pentose phosphate pathway (PPP) and acetyl-CoA production respectively. In addition, out of 7 regulated glucose transporters 4 were induced and 3 repressed by hypoxia. The results indicate mixed responses of glucose metabolizing genes. Decreased flux through the PPP is predicted to decrease antioxidant capacity through lower NADPH production, an effect consistent with the down-regulation of other anti-oxidant pathways discussed above.

### Hypoxia-related AS events

**Figure 7:**
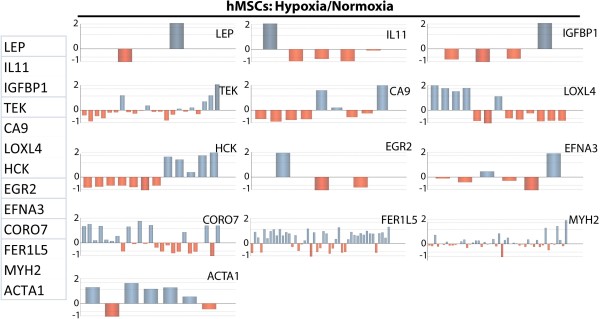
**Splicing maps of highly regulated genes with high splice indices.** Genes were selected based on highest positive (LEP, IL11, IGFBP1, TEK, CA9, LOX4, HCK, ERG2) and negative (EFNA3, CORO7, FER1L5, MYH2, ACTA1) fold change of gene expression hypoxia vs. normoxia. Bar graphs indicate hypoxic/normoxic differential exonic expression levels (n = 3).

Exon Microarrays identified 53 genes that were subjected to hypoxia-dependent AS; the arrays also revealed a correlation between high splice index and genes that responded the most strongly at the level of gene transcripts. As shown in Figure 7, eight of the most strongly induced genes and 5 of those that were most strongly repressed also had the highest splicing indices. This suggests a possible mechanistic link between gene regulation and control of AS by hypoxia. LEP, IL-11, IGFBP1, TEK, CA9, LOX4, HCK and EGR2 were each induced by more than 5-fold by hypoxia whereas EFNA3, CORO7, FER1L5, MYH2 and ACAT1 were each repressed (preferentially expressed in air) by greater than 4-fold under hypoxia. Four of the induced genes are HIF-1 targets and EFNA3 a down-regulated transcript, is an inhibitor of angiogenesis and target of the HIF-1-regulated microRNA-210 [57]. GO analyses indicated that the predicted functions of genes with hypoxia-regulated AS included cell adhesion, migration, apoptosis, angiogenesis and oxidation-reduction (Table 4). In terms of exon use, 8 of the identified AS events are described in the human genome annotation (5 C-terminus, 2 cassette-exon, 1 alt-3′ split site), and 16 involve constitutive exons. The remainder are novel AS events.

**Table 4:** GO enrichment classification of alternatively spliced genes after hypoxia

**Pathways**	**Gene symbol**	**Accession number**	**Splicing Index**	**Probe location**	**Gene description**
**Apoptosis-associated**					
Induction of apoptosis by intracellular signals	SART1	NM_005146	0.46	Exon11	Squamous cell carcinoma antigen recognized by T cells
Positive regulation of neuron apoptosis	PTPRF	NM_002840	2.56	Exon34	Protein tyrosine phosphatase, receptor type, F
Apoptosis	PPP1R13L	NM_006663	2.01	Exon13	Protein phosphatase 1, regulatory (inhibitor) subunit 13 like
	STEAP3	NM_182915	2.22	Exon6	STEAP family member 3
	TNFRSF14	NM_003820	2.39	Exon8	Tumor necrosis factor receptor superfamily, member 14 (herpesvirus entry mediator)
Negative regulation of apoptosis	ERCC2	NM_001130867	0.42	Exon8	Excision repair cross-complementing rodent repair deficiency, complementation group 2
Anti-apoptosis	SERPINB2	NM_002575	0.48	Exon8	Serpin peptidase inhibitor, clade B (ovalbumin), member 2
**Proliferation-associated**					
Cell growth	ACTA1	NM_001100	0.22	Exon6	Actin, alpha 1, skeletal muscle
	NDRG4	NM_001130487	2.62	Exon5	NDRG family member 4
Cell cycle checkpoint	ERCC2	NM_001130867	0.42	Exon8	Excision repair cross-complementing rodent repair deficiency, complementation group 2
Multicellular organism growth	ERCC2	NM_001130867	0.42	Exon8	Excision repair cross-complementing rodent repair deficiency, complementation group 2
Cell proliferation	ERCC2	NM_001130867	0.42	Exon8	Excision repair cross-complementing rodent repair deficiency, complementation group 2
	MT3	NM_005954	2.72	Exon3	Metallothionein 3
Cell cycle arrest	SART1	NM_005146	0.46	Exon11	Squamous cell carcinoma antigen recognized by T cells
Positive regulation of cell proliferation	IL11	NM_000641	7.99	Exon5	Interleukin 11
Negative regulation of cell proliferation	PTPRF	NM_002840	2.56	Exon34	Protein tyrosine phosphatase, receptor type, F
**Migration-associated**					
Axon guidance	SLIT3	NM_003062	0.44	Exon1	Slit homolog 3 (Drosophila)
	SEMA5A	NM_003966	2.90	Exon23	Sema domain, seven thrombospondin repeats (type 1 and type 1-like), transmembrane domain (TM) and short cytoplasmic domain, (semaphorin) 5A
Motor axon guidance	EGR2	NM_000399	5.08	Exon2	Early growth response 2
Cell migration	PVR	NM_001135770	0.49	Exon3	Poliovirus receptor
Positive regulation of cell migration	LAMB1	NM_002291	0.46	Exon9	Laminin, beta 1
Regulation of cell shape	ARAP1	NM_001135190	0.45	Exon5	ArfGAP with RhoGAP domain, ankyrin repeat and PH domain 1
**Angiogenesis-associated**					
Positive regulation of angiogenesis	RUNX1	NM_001122607	0.40	Exon1	Runt-related transcription factor 1
Wound healing	SERPINB2	NM_002575	0.48	Exon8	Serpin peptidase inhibitor, clade B (ovalbumin), member 2

Differentially spliced genes in human MSCs under hypoxia, were subjected to GO analysis as described in Methods.

**Figure 8:**
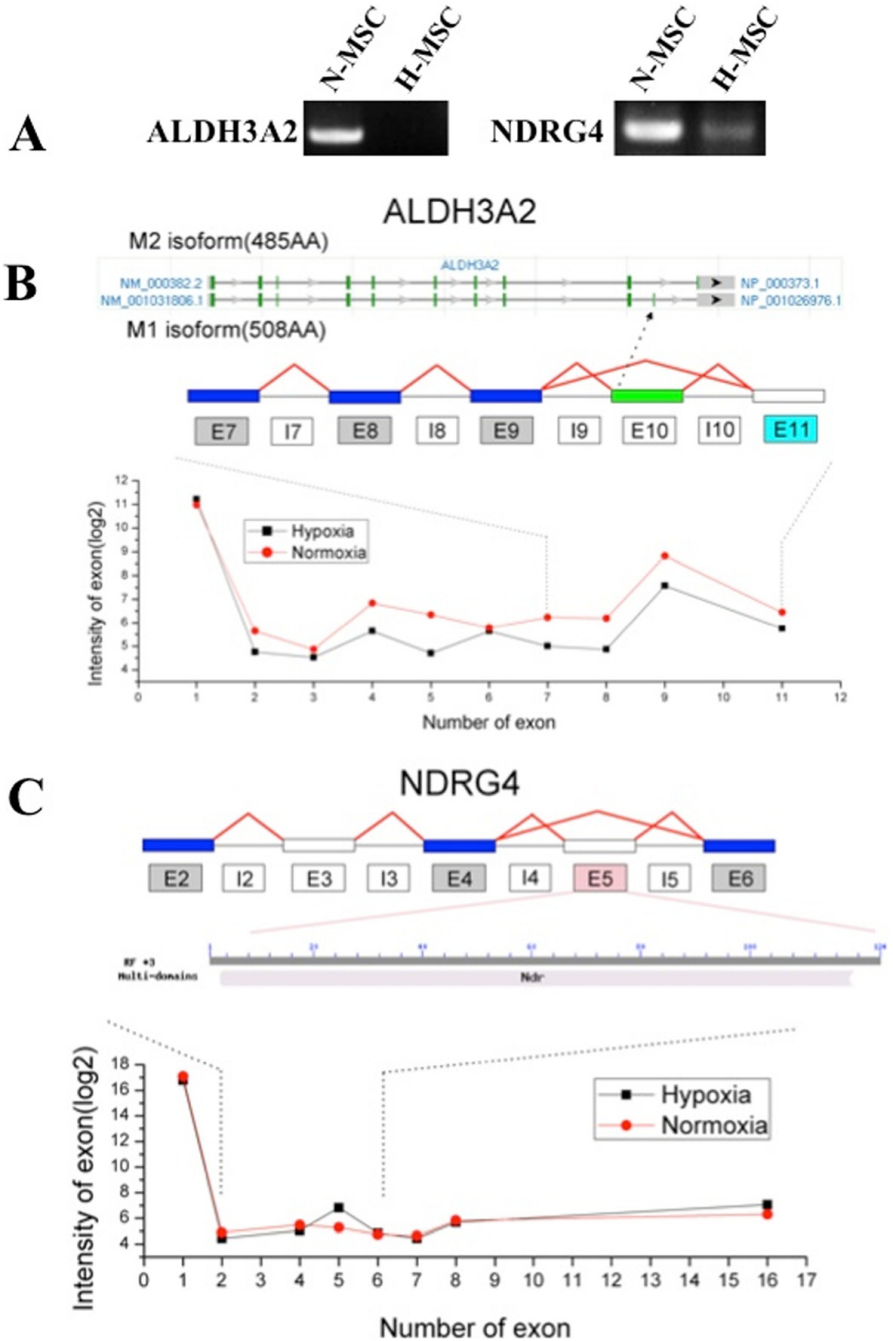
**Oxygen-dependent differential splicing of the ALDH3A2 and NDRG4 genes. (A)** Expression of splice isoforms confirmed by RT-PCR of genes with prior evidence of AS. **(B)** Annotation of each alternative isoform (M2 and M1, top graphic), exon structure (middle graphic) and expression profiles (bottom graphic) for ALDH3A2. Light blue boxes indicate down-regulation for hypoxic (H) versus normoxic (N) MSCs; gray boxes indicate no significant change; white boxes indicate no probe detection above expression threshold. The green exon (exon10) indicates the unique exon of isoform M1. Exon expression values (log2) are displayed for both H-MSC (black data points) and N-MSC (red data points), ranked in order of genomic position on the x-axis. The various isoforms can be validated in the PCR result although there is no probe annotated to the isoform-specific exon. **(C)** Exon structure (top graphic) modified domain of AS (middle graphic) and expression profiles (bottom graphic) for NDRG4. Light red boxes indicate up-regulation for hypoxic (H) versus normoxic MSCs (N) MSCs; gray boxes indicate no significant change; white boxes indicate no probe detection above expression threshold. A Modified or disrupted conserved domain of differentially expressed exon is from the CDD database (NCBI). Exon expression values (log2) are displayed for both H-MSC (black data points) and N-MSC (red data points), ranked in order of genomic position on the x-axis.

We chose two alternatively spliced genes, ALDH3A2 and NDRG4 for further analysis and qPCR confirmation; ALDH3A2 transcripts were decreased by hypoxia whereas NDRG4 were not regulated. ALDH3A2 is a member of the ALDH superfamily of NAD(P) + −dependent enzymes that catalyze the oxidation of a wide variety of aliphatic and aromatic aldehydes. ALDH3A2 is also known as fatty-aldehyde dehydrogenase (FALDH) because of its role in protecting against lipid peroxidation [58]; loss of ALDH3A2 activity is the cause of Sjogren-Larsson syndrome [59]. As shown in Figure 8A, hypoxia/normoxia dictate the use of AS to generate alternative isoforms of the ALDH3A2 gene. Exon array analysis revealed that the AS event occurred in the last exon, while PCR validation confirmed that the unique exon 10 was not expressed in hypoxic MSCs. This indicates preferentially expression of the M2 isoform under hypoxia (Figure 8B). Previous work has shown that hypoxia can repress the expression of both constitutive and induced ALDH3 isoforms [60] and it is also known that ALDH3A2 gene expression is positively regulated by PPARα [61]. Our finding that PPARα expression is also decreased by hypoxia in parallel with ALDH3A2 (Table 3) suggests a possible mechanism for the transcriptional repression of ALDH3A2 by hypoxia. Other work has shown that AS of the ALDH3A2 gene may determine its subcellular localization with physiological implications for function [62]. Our results are the first to show that hypoxia regulates AS of the FALDH gene.

The N-myc downstream-regulated gene (NDRG) family belongs to the alpha/beta hydrolase super family of genes that regulate survival, growth and differentiation of host cells. The NDRG4 isoform is preferentially expressed in brain and heart and its overexpression has been linked with an aggressive behavior of meningioma tumors [63]. AS variants of NDRG4 include heart (H) and two brain (B- and Bvar) isoforms [64]. As shown in Figure 8C, exon 5 of the NDRG4 gene was subject to hypoxia-dependent AS according to the exon microarray, and this was validated by qPCR. Alternatively spliced transcripts of the NDRG genes have been described although the functional significance is unknown [64,65].

### Tissue-specific regulation of gene expression and AS by hypoxia

Weigand et al. [35] recently reported results of their global analyses of hypoxia-regulated AS using human umbilical vein endothelial cells (HUVECs). Because our results as well as those of others suggest that many of the effects of hypoxia are tissue-specific we compared our gene expression and AS array data with that of Weigand et al. These results are shown in Supplement Figures 2, 3 and 4. Out of 446 and 113 genes regulated >2.5-fold by hypoxia in hMSCs and HUVECs respectively only 9 were commonly regulated including ANGPTL4, VEGFA, STC, SLC2A3, PGF, EGLN3, ADM, BHLHE40 and NQO1 (Additional file 2: Figure S2). Out of 541 and 239 gene isoforms created by hypoxia-regulated AS in hMSCs and HUVECs respectively, only 17 isoforms were common (Additional file 3: Figure S3). As shown in Additional file 4: Figure S4 comparing all of the genes in hMSCs and HUVECs regulated by hypoxia at the levels of gene expression and AS only 3 genes were common. These included Egln3, a prolyl hydroxylase involved in HIF-1 proteolytic targeting, stanniocalcin-1 (STC) a pro-angiogenic anti-apoptosis gene product [66,67] (both up-regulated) and NAD(P) Quinone oxidoreductase 1 (NQO1) a Phase II redox detoxification enzyme [68], (down-regulated). It should be noted that Weigand et al. exposed HUVECs to 1% oxygen for 48 h compared with our 0.5% for 24 h therefore the differences may be due in part to different oxygen tension as well as cell type.

## Discussion

Our studies suggest that severity of hypoxia within a range of 4–10 mmHg is a critical determinant of global gene expression that has important implications for the biology and function of human MSCs. Transcriptional profiling revealed altered patterns of gene expression particularly involving glucose metabolism, insulin/IGF-1 signaling, intermediate filaments, extracellular matrix, anti-oxidant enzymes, and differentiation-markers. The results are consistent with the quiescent, immobile state conferred by severe hypoxia along with reduced oxidative stress and a switch in favor of osteogenic, angiogenic and perhaps hematopoietic programs over those of adipogenesis and myogenesis. The gene expression profiles are distinct in many respects from those described previously for MSCs subjected to more moderate hypoxia that mediates enhanced proliferation [9,10,17-19].

Salient features of the present study that define the role of severe hypoxia and distinguish it from moderate hypoxia include the following: [1] Mixed responses of genes involved in glucose metabolism including the pentose phosphate and glycolysis pathways, and moderately induced or decreased levels of glucose transporter gene transcripts. Although glycolytic flux increased acutely under hypoxia, a lower than a maximal induction of all genes may be physiologically beneficial in the long term for a closed environment such as the endosteum where high glycolytic activity would generate localized acidosis and cell death. An analogous condition may be represented in anoxic turtles where the PGAM gene is also repressed and may contribute to the hypometabolic state that is necessary for the turtle to survive extended anoxia [69]. Notable also is the weak induction of Bnip3, a programmed cell death protein normally strongly induced by hypoxia through HIF-1α and co-regulated by hypoxia and acidosis [70]. Also noteworthy is the strong induction of carbonic anhydrase (CA9) suggesting a pre-emptive adaptation to acidosis [2]. IGF-1 and PI3-kinase transcripts were decreased while IGFBP-1, −3 and −5 transcripts were increased, and this was associated with depressed phosphorylation of Akt-Thr308 (Figure 6). The IGF-PI3-kinase pathway regulates cell growth and survival through key targets including mTOR and p70-S6-kinase, and its depressed activity by severe hypoxia is consistent with reduced growth and metabolism. PI3-kinase signaling and phosphor-Akt were reported to be activated in MSCs by moderate hypoxia [26] therefore the reduced activity by severe hypoxia again distinguishes the two conditions [6]. Acidic keratins were markedly increased by hypoxia and this correlated with a more polar organization of intermediate filaments and cell elongation. The acidic keratin genes are clustered on chromosome 17q and the coordinately increase levels of multiple transcripts suggests a common regulatory mechanism. Regulation of keratin expression by the HIF pathway has not been reported. It is also noteworthy that whereas acidic keratin transcripts were all induced, 3 basic keratin gene transcripts decreased. Cytosolic keratins are markers of epithelium and the strong inductions are reminiscent of mesenchymal to epithelial transition (MET) that is associated with reduced rates of growth and migration [43-45]. Such a partial transition is again consistent with the quiescent immobile state predicted by the severely hypoxic environment of the endosteum. Changes in expression of multiple collagen and actin genes provides additional evidence for dynamic changes in cytoskeleton and extracellular matrix that contribute to cell growth and mobility [3]. Reduced expression of antioxidant pathway genes in particular NAD(P)H quinone-1 and aldo-keto reductase family members is consistent with lower oxidative stress created by severe hypoxia and may be associated with lower electron leakage and free radical production by mitochondria. Lower ROS and oxidative stress may also contribute to reduced proliferation [4]. Enhanced expression of osteogenic and angiogenic marker genes and depressed expression of adipogenic and myogenic markers is consistent with some but not all previous studies. Mayer et al. [48] and Hung et al. [10] found that osteogenesis was induced in MSCs by an oxygen tension of 10 mmHg. Hung et al. also reported that adipogenesis was reduced by 10 mmHg whereas Fink et al. [46] and Kato et al. [16] reported the opposite effect with increased adipogenesis under the same pO_2_. The differences may be due to incubation conditions and whether the cell are exposed to reoxygenation during treatments; Hung et al. [12] reported that the effects of hypoxia on proliferation and differentiation were fully reversed by reoxygenation.

The induction of numerous markers of angiogenesis is consistent with many previous reports describing angiogenic activation by hypoxia. The selective decrease of multiple myogenic gene markers by severe hypoxia is a novel observation of this study.

This is the second report to describe global changes in the alternative splicing of genes mediated by hypoxia and the first to describe such an effect in stem cells. There was only a small amount of overlap of hypoxia-regulated alternatively spliced genes in HUVECs versus MSCs with only 17 commonly regulated exons. It is noteworthy that multiple genes that were highly regulated at the transcript level in hMSCs were also subject to differential exon inclusion suggesting possible common regulatory factors in both pathways. Regulators of AS include specific RNA binding proteins and transcription factors (reviewed in [32]). It is known that transcription factor binding can influence AS perhaps by influencing the concentration of direct AS regulators within the transcription complex, or by altering the rate of RNA polymerase II elongation, leading indirectly to AS. Extracellular signals can also modify AS events by changing the activity of regulators for example, hnRNP-A1 (heterogeneous nuclear ribonucleoprotein A1) an RNA binding protein involved in nuclear pre-RNA processing that inhibits the inclusion of multiple alternative exons becomes phosphorylated upon osmotic shock resulting in cytoplasmic accumulation with consequent effects on AS [71]. HIF-1 was shown to regulate differential splicing of the LDH gene in shrimps [72], as well as AS of the hTERT gene that regulates telomerase activity in mammalian cells [73]. Of the most strongly and dually (transcripts and AS) regulated genes LEP, IGFBP1, CA9 and LOXL4 are all HIF-1α regulated. Therefore HIF-1α may contribute to the AS of dependent genes providing dual and coordinated regulation of transcription and AS by hypoxia. It is difficult to assess the physiological significance of most of the AS responding genes without information on the functional consequences, however it was possible to group the genes into functional categories of cell survival, proliferation, mobility and differentiation (Table 4).

## Conclusion

We report on molecular genetic and phenotypic changes conferred on hMSC by severe hypoxia. The gene expression changes are largely distinct from those reported previously for more moderate hypoxia that support enhanced proliferation, and the results are consistent with a quiescent, immobile phenotype with reduced metabolic activity and lower oxidative stress. Hypoxia-mediated AS may contribute importantly to gene regulation and protein function during adaptation to a severely hypoxic environment such as that imposed by the bone marrow endosteum.

## Methods

### Isolation, culture and characterization of human MSC

Normal human bone marrow aspirates were obtained with written consent from healthy donors in accordance with the Declaration of Helsinki and with the approval of the Human subjects Ethics Committee of Second Affiliated Hospital of Zhejiang University. MSCs from 3 such donors were cultured as described previously [10,12,19,74]. Cell surface markers were profiled using a BD FACS CantoTM II Flow Cytometry System after 3–5 passages as described previously [75] with the following human specific monoclonal antibodies: CD29-phycoerythrin (PE) (eBioscience, San Diego, CA, USA), CD34-PE (MACS, Miltenyi Biotec, Auburn, CA, USA) and CD166-PE (BD Biosciences Pharmingen, San Diego, CA, USA), respectively.

### Hypoxia

MSCs were plated at 1 × 10^5^ cells/cm^2^ in complete culture medium and incubated under hypoxia (0.5% O_2_, 5% CO_2_) or normoxia (21% O_2_, 5% CO_2_) for 24 hours using a ProOX Model C21 system (BioSpherix, Redfield, NY, USA).

### RNA extraction

Total RNA from normoxic and hypoxic MSCs (3 independent donor samples each) was extracted using a Kit from Biochain, (Hayward, CA, USA), according to the manufacturer’s instructions. The RNA quality was assessed by formaldehyde agarose gel electrophoresis and quantified using a spectrophotometer (Nanodrop, Wilmington, DE, USA).

### RNA amplification and labeling

Gene expression microarray: RNA was amplified, reverse transcribed and labeled as described previously [36]. Briefly, 1 μg of total RNA was amplified using a Message AmpTM II RNA Amplification kit (Life Technologies, Austin, TX, USA). The RNA was reverse transcribed in the presence of cy3-dCTP or cy5-dCTP using Klenow enzyme. For exon microarray, RNA from 3 separate replicate samples of normoxic or hypoxia MSCs was amplified using a Low Input Quick Amp WT Labeling Kit from Agilent.

### Array hybridization and data acquisition

Hybridization, scanning and washing were performed on Agilent’s Microarray Platform according to standard protocols. Raw data were acquired using an Agilent DNA microarray scanner and Agilent feature extraction software. All data is MIAME compliant and raw files from the 6 arrays (n = 3 per group) are deposited in the GEO database at NCBI (GEO accession # GSE55875). In addition, we downloaded from the NCBI GEO database the 6 raw data files (n = 3 per group) from the AS study by Weigand et al. [35].

### Gene and AS analyses

All raw text files (from hMSC Agilent arrays) and CEL files (from HUVEC Affymetrix arrays) were imported into GeneSpring GX 11 software (Silicon Genetics, Redwood City, CA) for either global gene analysis or alternative splicing. A total of four analytical experiments were performed with 3 replicate arrays for each condition. Normalized expression values were calculated by the Robust Multi-array Average (RMA) method. The resultant signal information was analyzed using one-way analysis of variance (ANOVA) (p < 0.05), assuming normality and equal variances. Multiple Testing Correction (MTC) of p-values by Benjamini Hochberg screened out >90% of regulated genes (33/446) including many of the known HIF-1α-regulated genes such as metallothionein (MT3), carbonic anhydrase XII (CA12; 4.6-fold), (VEGFA; 4.6-fold) and placental growth factor, (PGF; 3.28-fold) (Table 1), as well as many of the gene transcripts that were subsequently confirmed by QPCR including phosphoglycerate kinase (PGK; 7.1-fold), phosphofructokinase (PFK; 3.2-fold), RAS p21 protein activator (RASA1; 2.9-fold) and Immediate Early Response 3 (IER3; 2.5-fold). MTC is known to screen out false positives as well as many true positives, therefore as in our previous micro-array studies [74,76] it was not employed here. The GeneSpring Cross Gene Error model was applied; this model determines the likelihood of observing a specific fold change to the likelihood of observing a fold measurement by the 50.0th percentile of all measurements in the sample. The average value of expression level for each gene across the samples is set to 1.0 and the resulting normalized signal value plotted for each sample. Lists of differentially expressed genes from different experiments were compared within GeneSpring and displayed as Venn diagrams to show overlapping and non-overlapping genes. Heatmaps and graphs were also generated within GeneSpring.

### Conserved domain function annotation of AS exons

To identify protein domains of genes modified by AS, a conserved domain database (CCD) from NCBI web services was used. By default, domain predictions are derived by comparing two protein isoform sequences: one that aligns to the alternative exon and another in which the exon is absent from the corresponding mRNA sequences (competitive isoform).

### Validation of the differentially expressed genes

Differentially expressed genes-of-interest were selected based on function and confirmed using quantitative real-time (RT-q) PCR. 2 μg of total RNA respectively from normoxic and hypoxic MSCs was reverse transcribed with oligo (dT)18 using M-MLV reverse transcriptase (TAKARA, Japan) in a volume of 40 μL. Following reverse transcription, 1 μl of this cDNA mixture was employed for a qPCR program of 40 cycles (10 s at 95°C/32 s at 60°C) with SYBR®Premix Ex TaqTM. Data were generated using Perfect Real Time (TAKARA, Japan) and Real-Time PCR (Applied Biosystems, Foster City, CA, USA). Data were analyzed by the 2-∆∆Ct method and results are shown as fold change relative to control.

### Confirmation of alternative exon expression

24 alternative exon sequences were selected for confirmation by RT-qPCR. The sequences of the AS exon and its neighboring exons were acquired from the Human Genome UCSC Genome Database. If AS of the last exon of a gene was indicated, the last two-exon sequences of this gene were accessed. Constitutive exon-specific primers were designed and after reverse transcription, qPCR products were separated in 1% agarose gels supplemented with ethidium bromide, and visualized by UV.

### Enzyme-linked immunosorbent assay (ELISA)

Leptin and VEGF concentrations in culture medium of normoxic and hypoxic MSCs were measured by ELISA (R&D System, Minneapolis, MN, USA), according to the manufacturer’s instructions. Assays were conducted on a SoftMax Pro® multiplate reader (Molecular Devices, Inc., Downingtown, PA, USA).

### Western blot

Our western blot procedures are described in detail elsewhere [77,78]. Briefly, equal amounts of protein were separated by 10-15% SDS polyacrylamide gel electrophoresis and electro-transferred onto an Immobilon-P Transfer Membrane (Millipore, Billerica, MA, USA). Membranes were blocked with 5% BSA in TBS-T and incubated with VEGF (Santa Cruz Biotechnology, Santa Cruz, CA, USA), leptin, α-tubulin, or Akt (R&D System), antibodies overnight at 4°C. Horseradish peroxide–conjugated secondary antibodies were hybridized by standard procedures. β-actin was used as loading control.

### F-actin and keratin intermediate filament staining

Normoxic and hypoxic MSCs were examined for actin filaments organization using Alexa Fluor 555 phalloidin (Invitrogen). Briefly, MSCs were fixed in 4% formaldehyde for 10 min, permeabilized with 0.1% Triton X-100 for 5 min, and blocked with PBS containing 1% BSA for 25 min. Cells were stained with diluted phalloidin in PBS for 20 min at room temperature and viewed under a fluorescence microscope.

### Statistical analysis

One-way ANOVA was used to compare experimental groups. Data are expressed as mean ± standard deviation (SD), and a p value < 0.05 was considered as statistically significant.

## Abbreviations

AS: Alternative splicing; MSC: Mesenchymal stem cell; MET: Mesenchymal to epidermal transition; VEGF: Vascular endothelial growth factor.

## Competing interest

The authors declare that they have no competing interests financial or non-financial related to the content of this article.

## Authors’ contributions

XH: conception, design of experiments and manuscript writing; RW: implement experiments, data analysis, manuscript writing; LS: data analysis, bioinformatics, statistics, manuscript writing; LZ, XH, FG, XL: implement experiments; HY: design of experiments and manuscript writing; KAW: conception, design of experiments, data analysis, manuscript writing, corresponding author; JW, conception, design of experiments, data analysis, manuscript writing, corresponding author. All authors read and approved the final manuscript.

## Supplementary Material

Additional file 1: Figure S1 Differential MSC gene expression. Heatmap of the top differentially expressed genes by at least 2.0-fold (p < .01) in hMSCs under hypoxia relative to normoxia. Click here for file

Additional file 2: Figure S2 Venn diagram and heatmap of differentially expressed genes that overlap in hMSCs and HUVECs under hypoxia. (A). Differentially expressed genes determined by our analyses of HUVECs and human MSCs under hypoxia were compared to find common differentially expressed transcripts. A 2.0 fold change and p < 0.01 significance cut-offs were used. (B). Selected genes, including VEGF-A, from the 9 transcripts overlapping in HUVEC and hMSCs, are shown by heatmap displaying expression levels in normoxic and hypoxic MSCs. Color bar shown is Log2. Click here for file

Additional file 3: Figure S3 Venn diagram and graphs of differentially expressed isoforms overlapping in hMSCs and HUVECs under hypoxia. (A). Differentially expressed isoforms determined by our analysis of HUVECs and human MSCs under hypoxia were compared to find common differentially expressed exons. A 1.0 splicing index and p < 0.01 significance cutoffs were used. (B). Exonic expression of 17 isoforms overlapping in HUVECs and hMSCs are shown in the graphs displaying the hypoxic vs. normoxic exonic expression levels. Click here for file

Additional file 4: Figure S4 Venn Diagrams and heatmap of differentially expressed genes overlapping genes in hMSCs versus HUVECs. Differentially expressed genes and isoforms determined by our analysis of HUVECs and human MSCs under hypoxia were compared to find common or unique differentially expressed genes and isoforms. A 1.0 splicing index, 2.0 fold change, and p < 0.01 significance cut-offs were used. Click here for file
