# Supplementary figures and images for: Severe hypoxia exerts parallel and cell-specific regulation of gene expression and alternative splicing in human mesenchymal stem cells
Source: BMC Genomics. 2014 Apr 23;15:303. doi: 10.1186/1471-2164-15-303 (PMC4234502; doi:10.1186/1471-2164-15-303)

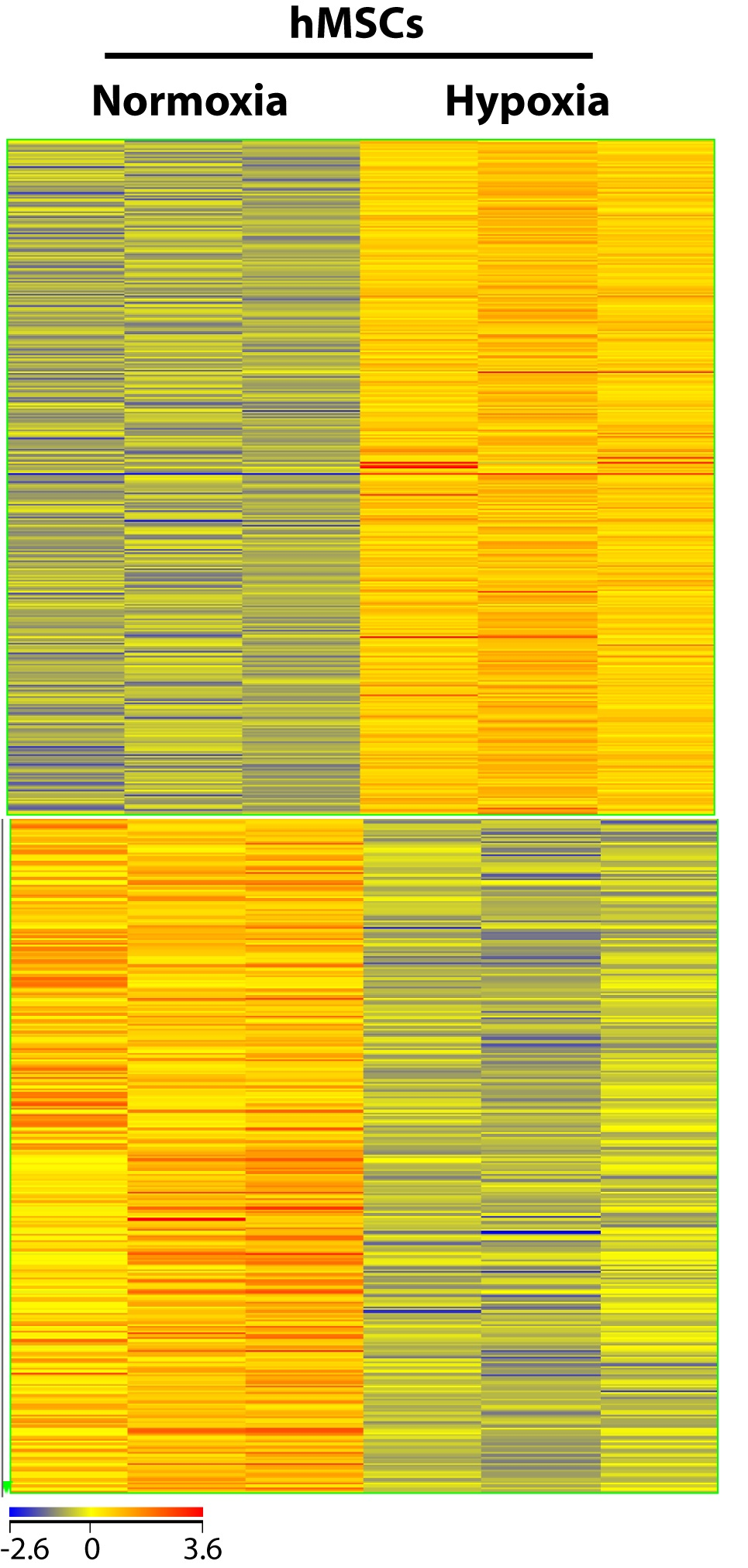

Supplement: Additional file 1: Figure S1 — Differential MSC gene expression. Heatmap of the top differentially expressed genes by at least 2.0-fold (p < .01) in hMSCs under hypoxia relative to normoxia. [file 1471-2164-15-303-S1.tiff]

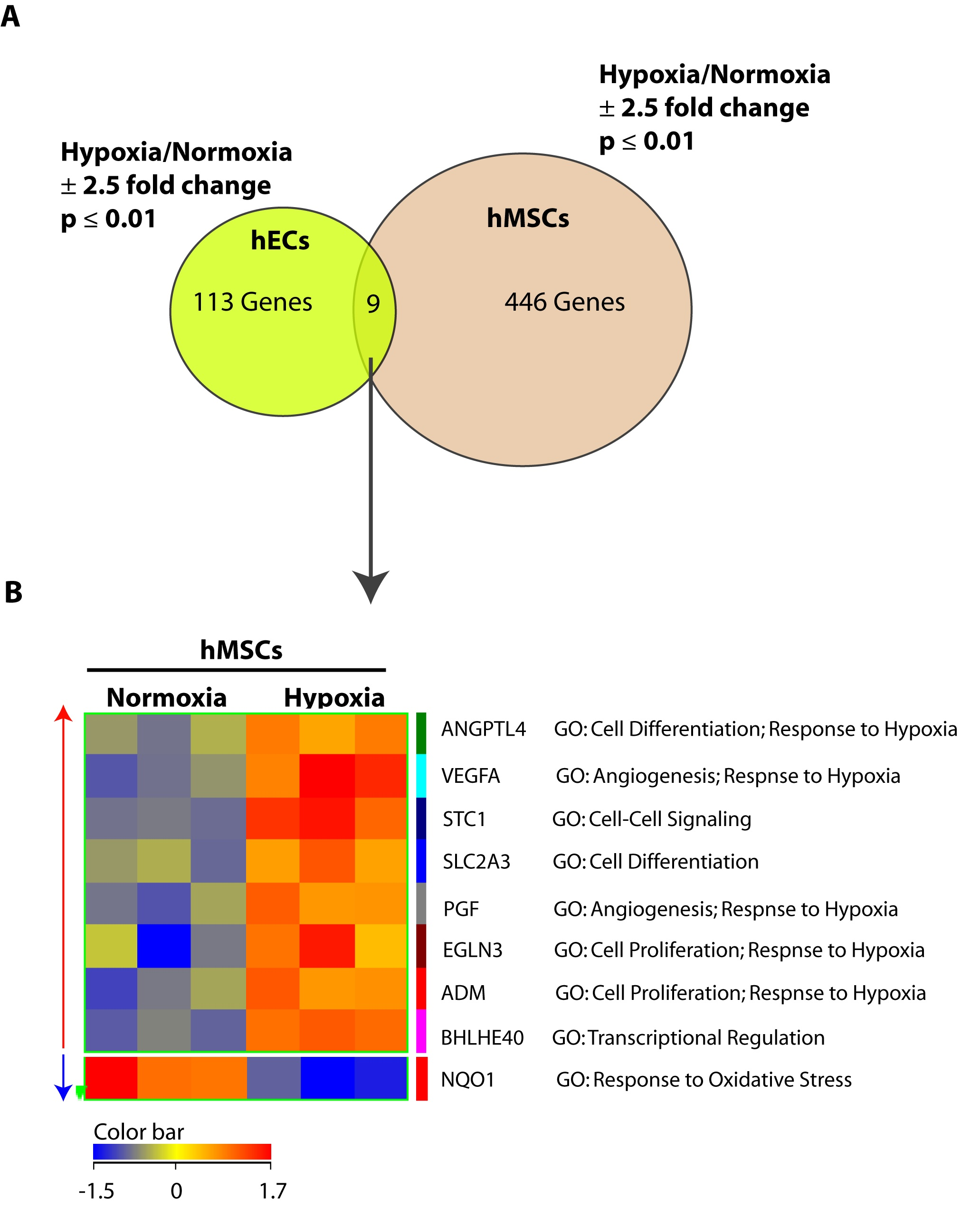

Supplement: Additional file 2: Figure S2 — Venn diagram and heatmap of differentially expressed genes that overlap in hMSCs and HUVECs under hypoxia. (A). Differentially expressed genes determined by our analyses of HUVECs and human MSCs under hypoxia were compared to find common differentially expressed transcripts. A 2.0 fold change and p < 0.01 significance cut-offs were used. (B). Selected genes, including VEGF-A, from the 9 transcripts overlapping in HUVEC and hMSCs, are shown by heatmap displaying expression levels in normoxic and hypoxic MSCs. Color bar shown is Log2. [file 1471-2164-15-303-S2.tiff]

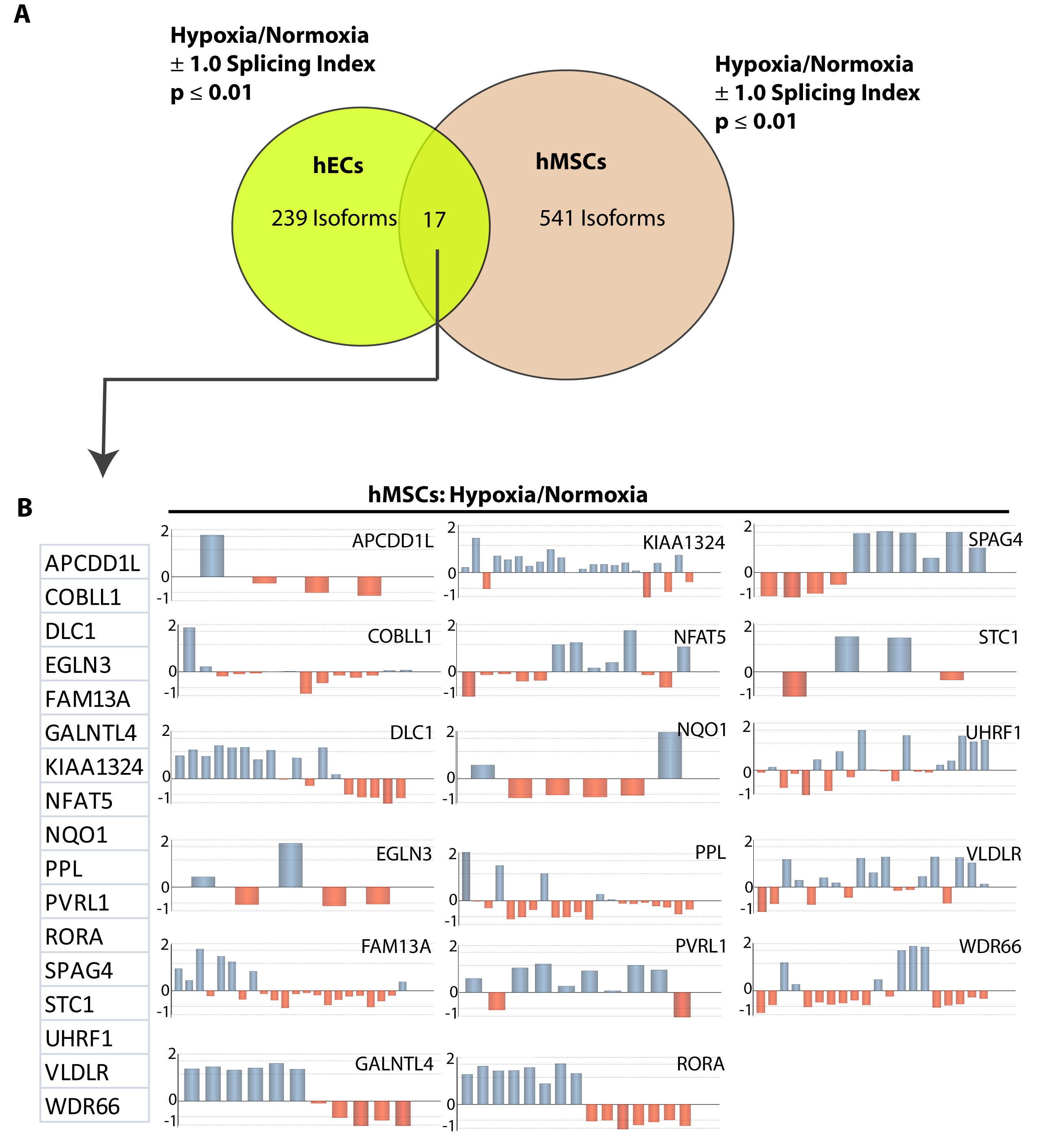

Supplement: Additional file 3: Figure S3 — Venn diagram and graphs of differentially expressed isoforms overlapping in hMSCs and HUVECs under hypoxia. (A). Differentially expressed isoforms determined by our analysis of HUVECs and human MSCs under hypoxia were compared to find common differentially expressed exons. A 1.0 splicing index and p < 0.01 significance cutoffs were used. (B). Exonic expression of 17 isoforms overlapping in HUVECs and hMSCs are shown in the graphs displaying the hypoxic vs. normoxic exonic expression levels. [file 1471-2164-15-303-S3.jpeg]

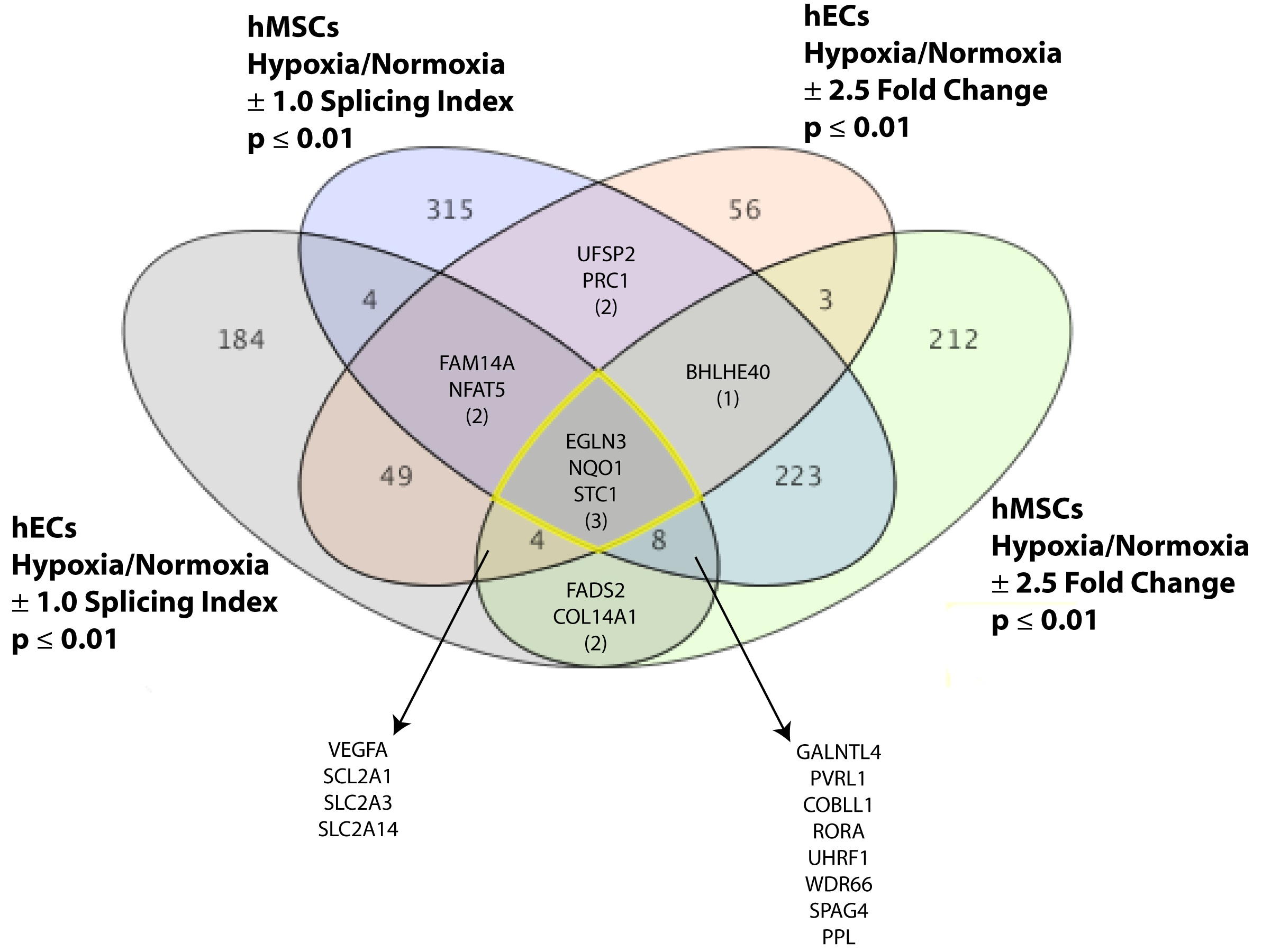

Supplement: Additional file 4: Figure S4 — Venn Diagrams and heatmap of differentially expressed genes overlapping genes in hMSCs versus HUVECs. Differentially expressed genes and isoforms determined by our analysis of HUVECs and human MSCs under hypoxia were compared to find common or unique differentially expressed genes and isoforms. A 1.0 splicing index, 2.0 fold change, and p < 0.01 significance cut-offs were used. [file 1471-2164-15-303-S4.tiff]
